# Child Linear Growth During and After the First 1000 Days Is Positively Associated with Intellectual Functioning and Mental Health in School-Age Children in Vietnam

**DOI:** 10.1093/jn/nxab182

**Published:** 2021-06-10

**Authors:** Phuong Hong Nguyen, Lan Mai Tran, Long Quynh Khuong, Melissa F Young, Thai Hong Duong, Hoang Cong Nguyen, Ann M DiGirolamo, Reynaldo Martorell, Usha Ramakrishnan

**Affiliations:** International Food Policy Research Institute, Washington, DC, USA; Thai Nguyen University of Pharmacy and Medicine, Thai Nguyen, Vietnam; Thai Nguyen National Hospital, Thai Nguyen, Vietnam; Hanoi University of Public Health, Hanoi, Vietnam; Hubert Department of Global Health, Emory University, Atlanta, GA, USA; Thai Nguyen University of Pharmacy and Medicine, Thai Nguyen, Vietnam; Thai Nguyen National Hospital, Thai Nguyen, Vietnam; Thai Nguyen University of Pharmacy and Medicine, Thai Nguyen, Vietnam; Thai Nguyen National Hospital, Thai Nguyen, Vietnam; Georgia State University, Atlanta, GA, USA; Hubert Department of Global Health, Emory University, Atlanta, GA, USA; Hubert Department of Global Health, Emory University, Atlanta, GA, USA

**Keywords:** child growth, intellectual functioning, mental health, PRECONCEPT, Vietnam

## Abstract

**Background:**

Millions of children fail to meet their developmental potential and experience mental health concerns globally. Evidence is mixed on whether growth beyond the first 1000 d of life influences intellectual functioning and mental health in school-age children.

**Objectives:**

We examined associations of childhood growth before and after the first 1000 d of life with child intellectual functioning and mental health at age 6–7 y.

**Methods:**

We used data from a follow-up of a randomized controlled trial of preconception supplementation (PRECONCEPT study) in Vietnam. A total of 5011 women participated in the study and 1579 children were born during 2012–2014. At age 6–7 y, child intellectual functioning was assessed using the Wechsler Intelligence Scale for Children, and mental health concerns were measured using the Strengths and Difficulties Questionnaire. Multivariable linear models were used to examine the independent association of child size at age 2 y [height-for-age *z*-score (HAZ) and body-mass-index *z*-score (BMIZ)] and conditional measures of linear and ponderal growth between the ages of 2 and 7 y.

**Results:**

HAZ at 2 y was positively associated with the Full-Scale Intelligence Quotient (*β* = 1.4; 95% CI: 0.5, 2.2 points) and its subdomains, namely Perceptual Reasoning Index, Working Memory Index, and Processing Speed Index (*β* = 1.0–1.4 points). Higher HAZ at 2 y was associated with lower overall mental health concerns (*β* = −0.24; 95% CI: −0.47, −0.01) and peer problems (*β* = −0.08; 95% CI: −0.17, −0.01). Faster height gain between 2 and 7 y was associated with higher total intellectual functioning (*β* = 0.9; 95% CI: 0.02, 1.8) and fewer emotional issues (*β* = −0.09; 95% CI: −0.18, −0.01). BMIZ at 2 y was not associated with intellectual functioning but was marginally associated with higher conduct and peer problems. Conditional weight gain between 2 and 7 y was not associated with child intellectual functioning or mental health in young school-age children.

**Conclusions:**

Child linear growth both during and beyond the first 1000 d is positively associated with intellectual functioning and mental health during the early school-age years.

## Introduction

Despite increasing global commitment and scaled-up support (1), suboptimal early childhood development continues to be a significant public health concern with an estimated 250 million children failing to achieve their full developmental potential (2). Additionally, 10–20% of children and adolescents worldwide experience poor mental health (3, 4). Multiple risk factors have been reported to influence brain development, cognitive functioning, school performance, and mental health. These include an array of psychosocial factors (such as caregiving competence, parental responsivity, maternal depression, and exposure to violence) and biological factors (such as maternal nutrition, prenatal and postnatal growth, nutrient deficiencies, infectious diseases, and environmental toxins) that have direct and indirect effects that begin from preconception through adulthood (5, 6).

The first 1000 d of life are recognized as a critical window of opportunity to influence human capital formation and are characterized by rapid growth and development (7). Most notably, the human brain has a growth spurt late in pregnancy and reaches 80% of adult size by the end of the second year, whereas remodeling, synaptogenesis, and other processes that are involved in higher-order tasks related to psychomotor and language development continue through childhood (7). Intrauterine growth restriction, mainly due to poor maternal nutrition before and during pregnancy, has been associated with delayed development during the early years as well as lower cognitive scores, and poorer learning and problem-solving ability later on (7). Similarly, faltering in linear growth during early childhood has been associated with poor development of language and motor skills at 18 mo (8), as shown in a study in Burkina Faso, Ghana, and Malawi (9). A meta-analysis of observational studies among low- and middle-income countries (LMIC) estimated that each 1 SD difference in length-for-age *z* score was associated with 0.28 SD and 0.24 SD difference in cognitive and motor development scores, respectively, in children aged <2 y (10).

The preschool and school years are periods during which considerable learning occurs and higher cognitive functioning and skills needed for success emerge (7, 11). It is a time of rapid and dramatic changes in the brain plasticity, and of fundamental acquisition of cognitive development and interpersonal skills (12). Both the learning environment and nutrition (including availability of protein, essential fatty acids, and micronutrients) during early childhood can influence brain functions such as neurogenesis, migration, cell division, myelination, and synaptic development (13–15), and thus influence later intellectual and mental health outcomes. Stunting, an indicator of linear growth retardation that has been estimated to affect >30% of children aged <5 y (16), has been associated with delays in cognitive ability at 5–11 y (10, 17), reduced school achievement, and increased risks of school dropout (18). Underweight and stunting were also associated with behavioral outcomes including more problems with conduct, poorer attention, and poorer social relationships at school age (19).

Although there are strong and consistent findings on the associations between growth in the first 1000 d and intellectual functioning or educational achievement (17, 20–22), evidence on the role of child growth beyond the first 1000 d is limited and contradictory. Some studies have reported positive associations of late postnatal growth with child development outcomes (20, 21), but others have not (23, 24). Other studies (25–29) have evaluated the role of pre- and postnatal growth, but lacked data on growth during the second year of life. Evidence on the relation between timing of growth and child mental health is also very limited; a study in Belarus showed that faster linear growth in the prenatal period and infancy was positively associated with mental health at early school age (30) whereas a large study in China did not observe these associations (31).

We have the unique opportunity to address the critical gaps in our understanding of the relative importance of timing of child growth on child intellectual functioning and mental health using a preconception longitudinal birth cohort in Vietnam. In this cohort, we have previously conducted a large randomized placebo-controlled trial (RCT) of preconception supplementation (32), and have shown that weekly supplementation with multiple micronutrients (MM) or iron and folic acid (IFA) improved linear growth and fine motor development at age 2 y (33). More recently, we also demonstrated that MM improved working memory and processing speed indices as well as overall intellectual functioning of offspring at 6–7 y (34) when compared with folic acid (FA). Our objective for the current study was to examine associations of summary measures of growth during the first 1000 d [as indicated by attained child size, height-for-age *z*-score (HAZ), and body-mass-index *z*-score (BMIZ), at 2 y] compared with later child growth (as indicated by conditional relative height and weight gain between 2 and 6–7 y) with child intellectual functioning and mental health at age 6–7 y. Findings from this study will inform the design of effective programs and policies that address the growing global health challenges of poor early childhood development, mental health concerns, and the double burden of malnutrition in many LMIC (35, 36).

## Methods

### Data sources and study population

Children in this study were offspring of mothers participating in an RCT (the PRECONCEPT study), which evaluated the effects of preconception micronutrient supplementation on maternal and child health outcomes in Vietnam (32). Details of the parent study have been published previously (32). Briefly, 5011 women of reproductive age were assigned randomly to receive weekly supplements containing either 2800 μg FA, 60 mg iron and 2800 μg FA (IFA), or MM containing the same amount of IFA, from baseline until conception, followed by daily prenatal supplements containing 60 mg iron and 400 μg FA until delivery. Women were followed prospectively to identify pregnancies and evaluate birth outcomes (1599 had live births, 10 twins). Live births were followed through age 2 y and at 6–7 y (**Supplemental Figure 1**) with follow-up rates of 97% and 91%, respectively. The current analysis includes 1392 children with available data on anthropometry, child intellectual development, and child mental health at 6–7 y.

### Outcome measures

At 6–7-y follow-up, child intellectual development was assessed using the Wechsler Intelligence Scale for Children—Fourth Edition (WISC-IV) (37). The WISC-IV consists of 10 core subtests: Vocabulary, Similarities, Comprehension, Block Design, Picture Concepts, Matrix Reasoning, Digit Span, Letter-Number Sequencing, Coding, and Symbol Search. These are summed into 4 indices that represent intellectual functioning in 4 specific cognitive domains [Verbal Comprehension Index (VCI); Perceptual Reasoning Index (PRI); Working Memory Index (WMI); and Processing Speed Index (PSI)] and the Full-Scale Intelligence Quotient (FSIQ). This test has been validated and adapted in the Vietnamese context, including the translation, cultural analysis, modifications, and standardization (38). The WISC-IV was administered following standard procedures (34) by pediatricians or researchers with a master's degree in public health after 2-wk extensive training including classroom lectures and discussions, mock interview, field practice, and debriefing. Weekly field-based supervision was used for quality control of the assessment. Refresher training sessions were conducted every 6 mo after the initial training to ensure testing was conducted in a standardized manner.

Child mental health was assessed using the Strengths and Difficulties Questionnaire (SDQ) (39), which was administered to the child's primary caregivers. The SDQ consists of 25 core items, each rated as not true (0), somewhat true (1), or certainly true (2). The scores from these items were used to construct 5 subscales (Emotional Symptoms, Conduct Problems, Hyperactivity, Peer Problems, and Prosocial Behavior), each ranging from 0 to 10; the first 4 subscales were summed to generate a total difficulty scale (range from 0 to 40), with higher scores indicating greater mental health concerns. The tool has been validated and widely used to assess emotional and behavioral disorders in children aged 4 to 16 y in several countries including Vietnam (40, 41).

### Exposure variables

Child anthropometry was collected at various ages (birth, 6 mo, 1, 2, and 6–7 y) by trained field staff using standard methods (42). Child weight was measured using electronic weighing scales precise to 10 g, and child length/height was measured with collapsible length boards, which were precise to 1 mm. The average of duplicate measurements of height and weight were then converted into HAZs and BMIZs according to 2006 WHO child growth standards (43).

Because we aimed to test whether growth during the first 1000 d or beyond is important, we used *1*) child attained size at age 2 y (HAZ and BMIZ) as a summary indicator of child growth during the first 1000 d, which includes growth in utero and the first 2 y of life, and *2*) conditional measures of linear and ponderal growth from 2 y to 6–7 y as the key exposure variables. Because linear growth and weight gain are highly correlated and we have repeated measures over time for each child, we developed the child conditional growth measures to produce uncorrelated height and weight gain for the 2 to 6–7-y window. Conditional relative height/weight gain between 2 and 7 y was computed as the standardized residuals from linear regressions of anthropometric measures at present (i.e., 7 y) on all prior measures (i.e., at 2 y). The conditional height gain was the present height accounting for height, and weight at 2 y. Conditional relative weight gain was the present weight accounting for present length and all previous weight and length measures. Because the conditional relative height/weight gain is the height/weight deviation from the child's previous growth trajectory, it represents the relative speed of length/weight gains during an interval of childhood. This method has been widely used in previous studies (20, 22, 39, 44) and allows us to compare the influence of linear growth and relative weight gain on outcomes. In supplemental analysis, we also examined models that partitioned growth during the first 1000 d into prenatal and postnatal periods by including measures of birth size (birth length *z*-score; birth weight *z*-score) and conditional length/weight gain for 0–2 y and 2–7 y.

### Confounders

Confounding variables were considered at child, caregiving, maternal, and environment levels, building from prior conceptual models (6, 19). Child-level variables included child age, sex, and birth outcomes [preterm birth (<37 wk of gestation), low birth weight (<2500 g), and small for gestational age (SGA), which was defined as a birth weight <10th percentile for gestational age (45)]. Child morbidity was collected through maternal recall of symptoms (fever, cough, flu, diarrhea) in the 2 wk prior to the survey (46, 47). Caregiving-level variables included child feeding practices such as breastfeeding and complementary feeding. Child dietary diversity was assessed as number of food groups consumed at ages 2 y and 6–7 y using the standard WHO guidelines (48), based on the maternal recall of all foods and liquids given to children in the last 24 h prior to the survey. Maternal-level variables included education, nutritional status, depressive symptoms, and intellectual ability, which were assessed at baseline. Maternal education was categorized into 4 groups: primary school (completed 1–5 y), secondary school (6–9 y), high school (10–12 y), and college or higher. Preconception anemia was defined as hemoglobin (measured by HemoCue with a finger-prick capillary blood sample) <12 g/L. Maternal depressive symptoms were measured at community health centers by trained psychologists using the Center for Epidemiologic Studies Depression Scale (49), which has been adapted for use in Vietnam (50). Finally, maternal intellectual ability was assessed using the Raven Progressive Matrices IQ Test (51) at the participant's home by well-trained researchers. Environmental-level variables included child learning environment and household socioeconomic status (SES). Child enrollment in daycare centers at the age of 0–36 mo, or in preschools at age 36–72 mo, was used to assess the early childhood learning environment. The quality of the learning environment at home was measured by field researchers using the Infant/Toddler HOME (Home Observation for Measurement of the Environment) Inventory at 1 y, and the Middle Childhood HOME Inventory at 6–7 y (52); the HOME assesses the quality and quantity of the social, emotional, and cognitive support available to a child in the home environment. These instruments were translated to Vietnamese, back-translated, and verified for construct validity during pretesting sessions. Results from our standardization showed high interrater reliability with coefficients >90% for various HOME subscales. Household SES index at baseline was calculated using a principal components analysis of housing quality and ownership of assets, and then categorized into quartiles (53).

### Statistical analysis

Normality of the continuous outcome variables was assessed using the Kolmogorov–Smirnov test. Descriptive analyses (means, SDs, percentages) were used to report characteristics of the study population. Multivariable linear regressions were used to assess the association of early childhood growth with intellectual development and mental health at school age (both with overall scores and with each subdomain). Associations with each outcome were examined in 2 models: *1*) adjusted only for child age and sex; and *2*) fully adjusted for selected child (age, sex), maternal (age, parity, education, depressive symptoms and treatment group), caregiving, and environmental factors (preschool education, home environment at 1 y and 6–7 y, household SES). Results from these models were expressed as differences in outcomes associated with a 1 SD change in child size at 2 y or in the conditional variables. We also examined the interaction between early child growth and child sex, household SES, and home environment on outcomes. All data analyses were performed using Stata version 16 (StataCorp LLC).

### Ethical approval

The study was approved by the Ethical Committee of the Institute of Social and Medicine Studies in Vietnam and Emory University's Institutional Review Board, Atlanta, Georgia, USA. The trial was registered in the US Clinical Trials registry (identification number NCT01665378). Written informed consent was obtained from all study participants.

## Results

The final study sample included all singleton live births born to women with available data at 6–7 y on offspring anthropometry, intellectual functioning (*n *= 1267), and mental health (*n *= 1392) (Supplemental Figure 1). At 6–7 y, we lost ∼3% (*n *= 42) of the cohort due to migration out of the study area (*n *= 29), drop out of study (*n *= 5), or child death (*n *= 8). The most common reason for missing data was that participants did not attend the visits (children were sick, or caregivers were not available to bring children to community health centers during the study time). The final analytic sample was similar on most baseline characteristics to those with missing data (**Supplemental Table 1**).

**Table 1** shows selected maternal characteristics that were measured at the time of recruitment (preconception), child characteristics, and the quality of home environment at ages 1 and 6–7 y. The mean age at follow-up was 6.4 y, and nearly half of the sample were girls (49.5%). At birth, 10% of children were preterm, 5% were low birthweight, and 15% were SGA. The descriptive statistics for the key outcomes and their subscales as well as measures of child attained size and nutritional status are presented in **Table 2**. Mean FSIQ was 88.2 ± 12.2, and mean subdomains of intellectual functioning ranged from 81.7 ± 12.5 for VCI to 101.7 ± 11.6 for WMI. Mean total SDQ was 7.4 ± 3.4, and the mean scores for the different subcomponents were ∼1 for emotional symptoms and conduct problems, 1.5 for peer problems, 3.8 for hyperactivity, and 6.7 for prosocial behavior.

**FIGURE 1:**
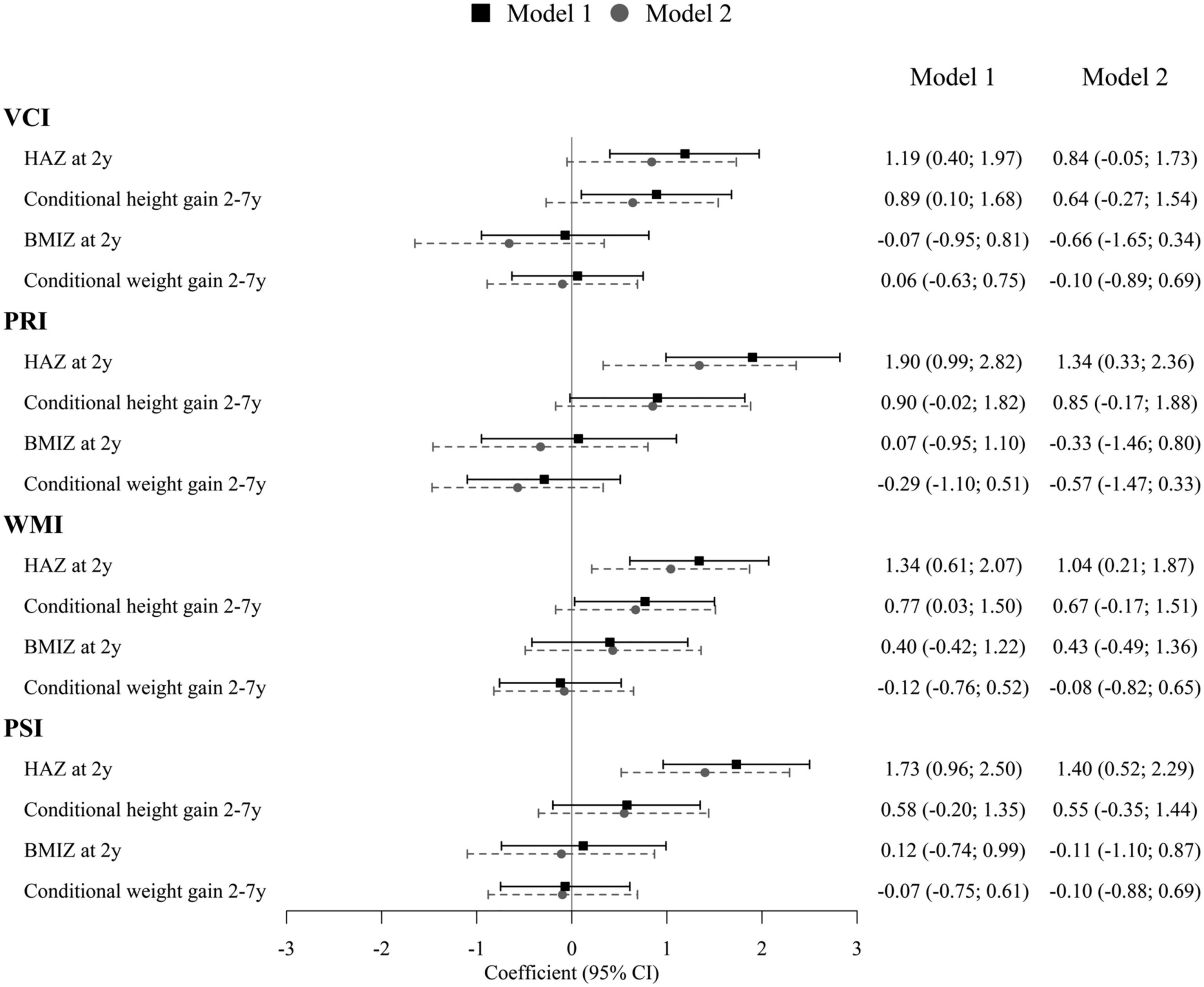
Associations of child growth during the first 1000 d and beyond with 4 specific cognitive domains of intellectual functioning at 6–7 y. Model 1 adjusted for child age and sex; model 2 adjusted for maternal (age, parity, and education) and child factors (age, sex, and preschool education), home environment at 1 y and 6–7 y, household socioeconomic status, and treatment group. BMIZ, body-mass-index for-age *z*-score; HAZ, height-for-age *z*-score; PRI, Perceptual Reasoning Index; PSI, Processing Speed Index; VCI, Verbal Comprehension Index; WMI, Working Memory Index.

**TABLE 1:** Description of study population^1^

	Study population
	*n* = 1392
Maternal characteristics	
Age, y	25.9 ± 4.3
Education, %	
Primary school	7.5
Middle school	55.3
High school	25.6
College or higher	11.7
Depressive symptoms (CES-D ≥10), %	9.2
Maternal IQ, score	86.7 ± 16.9
Child characteristics	
Child age at follow-up, y	6.4 ± 0.4
Female, %	49.5
Gestational age, wk	39.2 ± 2.0
Preterm, %	9.6
Exclusive breastfeeding at 3 mo, %	59.5
Enrolled in preschool during 0 to <36 mo, %	34.9
Enrolled in kindergarten during 36–72 mo, %	99.9
Morbidity in the previous 2 wk (measured at 6–7 y), %	
Fever	12.7
Pneumonia/severe cough	14.6
Flu	13.4
Diarrhea	1.1
Dietary diversity score at 2 y, score	4.7 ± 1.2
Dietary diversity score at 6–7 y, score	5.5 ± 1.5
Household characteristics	
Home environment at 1 y, score	63.3 ± 8.2
Home environment at 6–7 y , score	55.4 ± 13.7

1 Values are means ± SDs or percentages. CES-D, Center for Epidemiologic Studies Depression Scale; IQ, Intelligence Quotient.

**TABLE 2:** Summary statistics of child intellectual functioning, mental health, and nutritional status^1^

	Study population
	*n* = 1392
Child intellectual functioning at 6–7 y, score	
VCI	81.7 ± 12.5
PRI	93.1 ± 14.5
WMI	101.7 ± 11.6
PSI	89.3 ± 12.3
FSIQ	88.2 ± 12.2
Child mental health at 6–7 y,^2^ score	
Emotional symptoms	1.1 ± 1.3
Conduct problem	1.1 ± 1.1
Hyperactivity	3.8 ± 1.4
Peer problem	1.5 ± 1.2
Prosocial behavior	6.7 ± 1.9
SDQ score	7.4 ± 3.4
Child size and nutritional status	
At birth	
Low birth weight, %	4.8
SGA, %	15.3
LAZ	−0.30 ± 1.5
WAZ	−0.32 ± 1.6
At 24 mo	
Weight, kg	9.8 ± 1.0
Length, cm	78.0 ± 2.7
HAZ	−1.1 ± 0.9
BMIZ	−0.05 ± 0.8
Stunting, %	16.4
Overweight/obese, %	9.6
At 6–7 y	
Weight, kg	18.8 ± 3.2
Height, cm	113.6 ± 5.3
HAZ	−0.8 ± 0.9
BMIZ	−0.7 ± 1.1
Stunting, %	9.6
Overweight/obese, %	6.8

1 Values are means ± SDs or percentages. BMIZ, body-mass-index for-age *z*-score; FSIQ, Full-Scale Intelligence Quotient; HAZ, height-for-age *z*-score; LAZ, length-for-age *z*-score; PRI, Perceptual Reasoning Index; PSI, Processing Speed Index; SDQ, Strengths and Difficulties Questionnaire; SGA, small for gestational age; VCI, Verbal Comprehension Index; WAZ, weight-for-age *z*-score; WMI, Working Memory Index.

2 SDQ score was calculated from the sum of 4 subscales (Emotional Symptoms, Conduct Problems, Hyperactivity, and Peer Problems); for each subscale and total score, the higher scores indicate greater mental health concerns. For Prosocial Behavior, higher scores indicate more positive behavior.

HAZ at 2 y was significantly and positively associated with overall intellectual functioning (**Table 3**) as well as the different developmental subdomains at age 6–7 y (**Figure 1**). A 1 SD higher HAZ at 2 y was associated with FSIQ increased by 1.4 points (95% CI: 0.5, 2.2), PRI by1.3 points (95% CI: 0.3, 2.3), WMI by 1.1 points (95% CI: 0.3, 1.9), and PSI by 1.4 points (95% CI: 0.6, 2.3). Faster height gain between 2 and 7 y was also positively associated with intellectual functioning and remained significant only for total FSIQ (*β* = 0.9; 95% CI: 0.02, 1.8) after adjusting for other confounders. No association was observed between child intellectual functioning and BMIZ at 2 y or conditional weight gain between 2 and 7 y. Other factors that were positively associated with FSIQ included maternal education, higher quality of HOME environment at 12 mo, and higher SES. Child feeding variables were not statistically significant, and inclusion of maternal intellectual ability (IQ), which was correlated with maternal education, did not significantly alter findings. There is no evidence of heterogeneous effects by child sex, household SES, and home environment (data not shown). The additional findings when we divided growth during the first 1000 d into fetal (birth length *z*-score) and postnatal components showed that conditional measures of height gain from 0 to 2 y had an attenuated coefficient (0.96; 95% CI: 0.10, 1.81) compared with that of HAZ at 2 y (1.36; 95% CI: 0.50, 2.21) on FSIQ (**Supplemental Table 2**).

**TABLE 3:** Associations of child growth during the first 1000 d and beyond with child intellectual development and mental health at 6–7 y^1^

	FSIQ (*n *= 1267)		SDQ (*n* = 1392)	
	Model 1	Model 2	Model 1	Model 2
Outcomes	*β* (95% CI)	*β* (95% CI)	*β* (95% CI)	*β* (95% CI)
HAZ at 2 y	1.87 (1.10, 2.63)	1.36 (0.50, 2.21)	−0.26 (−0.46, −0.05)	−0.23 (−0.46, −0.01)
Conditional relative height gain between 2 and 7 y	1.04 (0.27, 1.81)	0.89 (0.02, 1.75)	−0.24 (−0.44, −0.04)	−0.17 (−0.40, 0.05)
BMIZ at 2 y	0.14 (−0.72, 1.00)	−0.26 (−1.22, 0.69)	0.23 (0.01, 0.46)	0.21 (−0.04, 0.46)
Conditional relative weight gain between 2 and 7 y	−0.13 (−0.80, 0.54)	−0.28 (−1.04, 0.48)	−0.07 (−0.25, 0.11)	−0.11 (−0.31, 0.09)

1 Values are means ± SDs or percentages. SDQ score was calculated from the sum of 4 subscales (Emotional Symptoms, Conduct Problems, Hyperactivity, and Peer Problems); for each subscale and total score, the higher scores indicate greater mental health concerns. Model 1 adjusted for child age and sex; model 2 adjusted for maternal (age, parity, and education) and child factors (age, sex, and preschool education), home environment at 1 y and 6–7 y, household socioeconomic status, and treatment group. BMIZ, body-mass-index for-age *z*-score; FSIQ, Full-Scale Intellectual Quotient; HAZ, height-for-age *z*-score; SDQ, Strengths and Difficulties Questionnaire.

**FIGURE 2:**
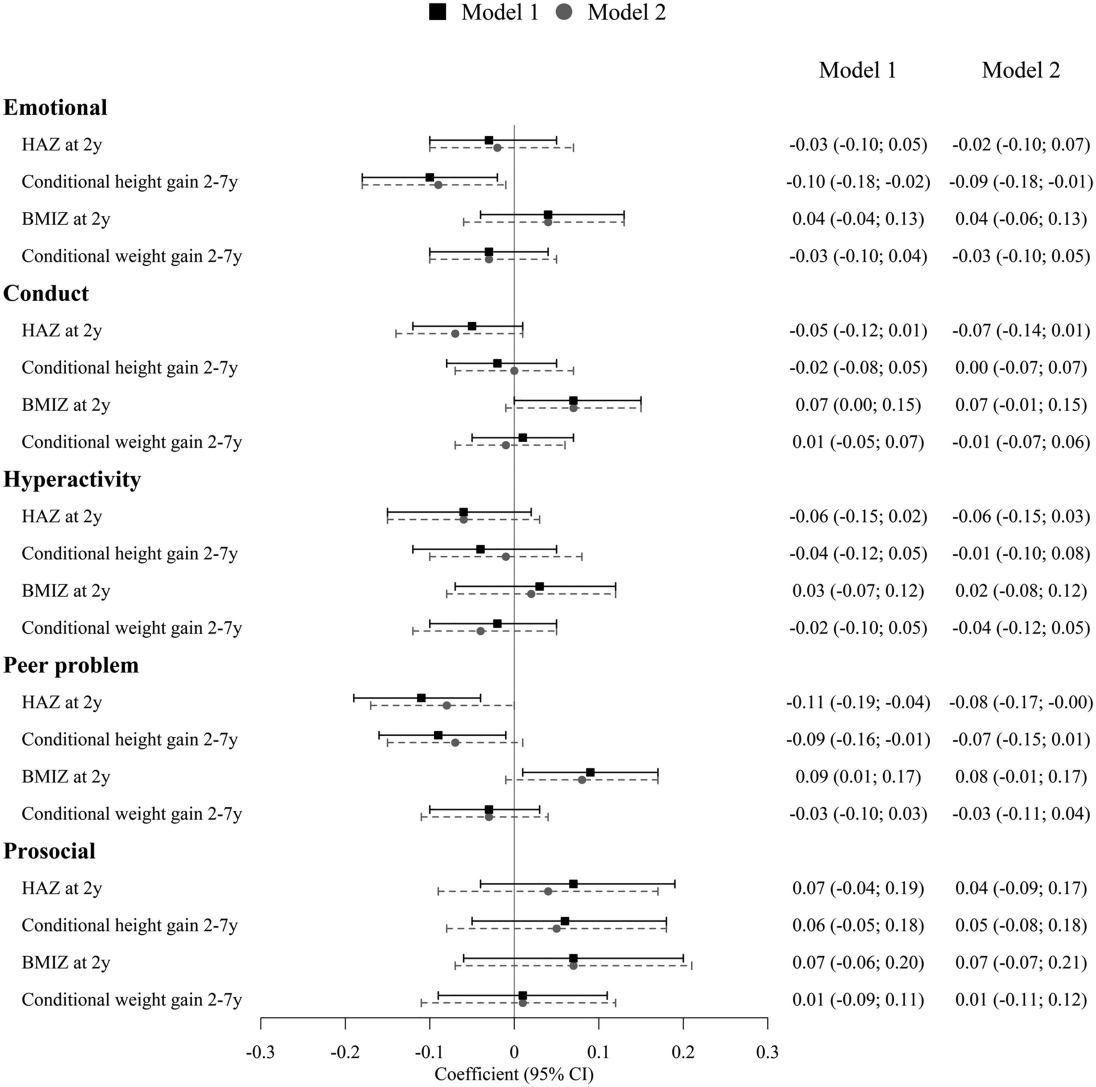
Associations of child growth during the first 1000 d and beyond with 5 subscales of mental health at 6–7 y. Model 1 adjusted for child age and sex; model 2 adjusted for maternal (age, parity, and education) and child factors (age, sex, and preschool education), home environment at 1 y and 6–7 y, household socioeconomic status, and treatment group. For each subscale of mental health, higher scores denote more mental health concerns except for prosocial where higher scores indicate more positive behavior. BMIZ, body-mass-index for-age *z*-score; HAZ, height-for-age *z*-score.

For child mental health, higher HAZ at 2 y was associated with lower scores for overall mental health concerns (*β* = −0.24; 95% CI: −0.47, −0.01) (Table 2) and peer problems (*β* = −0.08; 95% CI: −0.17, −0.01). Faster height gain between 2 and 7 y was associated with lower scores on the emotional symptoms (*β* = −0.09; 95% CI: −0.18, −0.01) (**Figure 2**), signifying a positive association between child growth and mental health. Higher BMIZ at 2 y was associated with higher conduct and peer problems, but these associations were marginally statistically significant after adjusting for confounding variables (*P *= 0.08). Conditional weight gain between 2 and 7 y was not associated with mental health. Other factors that were positively associated with better mental health included being a girl and higher home environment scores at 6–7 y (data not shown).

## Discussion

Our findings show the importance of growth both during the first 1000 d and thereafter for optimizing intellectual functioning and mental health in young school-aged children in Vietnam. HAZ at 2 y was positively associated with total intellectual functioning and its subdomains, and negatively associated with overall mental health concerns and peer problems in children aged 6–7 y. Faster height gain between 2 and 7 y was also associated with higher total intellectual functioning and fewer emotional issues. Although the coefficients for HAZ at 2 y were consistently larger and more precisely estimated than for linear growth for 2–7 y, we cannot conclude that the magnitude of the associations differed significantly because the CIs overlapped substantially. In contrast, both BMIZ at 2 y and conditional weight gain between 2 and 7 y were not associated with intellectual functioning. However, BMIZ at 2 y was marginally associated with higher conduct and peer problems.

Our findings confirm previously reported positive associations between child linear growth in the first 1000 d through school age and later intellectual functioning. We used attained child size at 2 y as a summary measure of growth during the first 1000 d (i.e., from conception through age 2 y), and have previously demonstrated that there is linear growth failure both in utero (54), as shown by the negative *z*-score for birth length (LAZ = −0.32) and postnatally when HAZ continues to decline further to age 2 y (HAZ = −1.1). When we split the first 1000 d into fetal and postnatal components, birth length *z*-score was not associated with FSIQ but conditional height gain from 0 to 2 y remained significant, although it had an attenuated coefficient when compared with that of HAZ at 2 y. Some studies, however, have shown stronger results for growth in the first 2 y when compared with later growth up to early school age. In a Filipino cohort, increases in cognitive ability were larger for changes in HAZ from 6 to 24 mo than from 2 to 11 y (21). In Thailand, growth up to 1 y of age but not from 1 to 9 y was associated with intellectual functioning at age 9 y (24). Finally, findings from Guatemala showed that nutritional supplementation during the first 1000 d but not later increased years of schooling, scores on reading comprehension, and IQ during adulthood, 25 y after the intervention ended (55). However, our findings suggest that growth beyond 2 y up to early school age is also important for intellectual functioning. Several reports from the Young Lives study (25, 26, 28, 29), which was conducted in Ethiopia, India, Peru, and Vietnam, also found similar associations. Both HAZ at ∼1 y and conditional growth between ∼1 and 8 y were positively associated with mathematics achievement, reading comprehension, and receptive vocabulary (26). Children who recovered in linear growth from age 1 to 8 y also had better outcomes when compared with those who were persistently stunted during this period (24). A limitation of these studies, however, is that the measures for later growth include substantial growth during the first 1000 d because the measure representing the first 1000 d was taken between 6 and 18 mo of life.

Our study contributes to the limited evidence on the importance of child growth during the early years for later mental health. Results from a previous study in Belarus showed that both weight and length gain in infancy were negatively associated with externalizing behaviors and positively associated with prosocial behavior (30). Our findings confirmed that linear growth in the first 1000 d was positively associated with better mental health and fewer peer problems, and provided additional evidence on the associations of higher linear growth beyond the first 1000 d with fewer emotional issues. We also observed that higher BMIZ at 2 y might be associated with higher conduct and peer problems; these findings are aligned with other research that suggests that higher BMI and child overweight/obesity in school-age children can be associated with psychological and psychosocial effects such as body image disturbances, low self-esteem, impaired social relationships, high levels of internalizing (depression and anxiety), and externalizing behavioral problems (hyperactivity and aggressiveness) (56). However, there are limited data in young children prior to school in LMIC, and further research is needed to better understand underlying drivers for these associations. Given the emerging double burden of malnutrition in many LMIC that have undergone a nutrition transition (16, 36, 57), including Vietnam, optimizing targeted interventions that reduce the burden of child undernutrition yet prevent overweight/obesity might also help to improve child mental health.

A few methodological limitations are noted in this study. Although we adjusted for a wide range of maternal and child characteristics, residual confounding by genetic and environmental factors cannot be excluded. We had data on when children started daycare and the total time they spent in daycare, but lacked data on the quality of the early childhood learning environment in daycare settings and/or preschool. Child feeding was assessed by a single point 24-h dietary recall, which could be unrepresentative of overall dietary exposure. Loss to follow-up due to migration or lack of interest could have also caused nonresponse bias.

Major strengths of this study include the availability of prospectively collected data from a well-characterized mother-child cohort that spans from preconception, during pregnancy and early childhood in low-resource settings with a high follow-up rate at the age of 6–7 y, combined with comprehensive measures of intellectual functioning and its subdomains, total mental health concerns as well as specific subscales, and the quality of the learning environment during early childhood. All the measurement tools have been validated and well adapted for the local context (38, 40). Our large sample size, high rates of follow-up, and availability of child growth measurements exactly at 2 y, allow us clearly to separate the influence of linear and ponderal growth during the first 1000 d and thereafter on child intellectual functioning and mental health at school age. Our analytical approach using conditional measures of growth also allowed us to avoid collinearity between multiple measurements and separate the roles of linear growth from soft tissue gain (20).

In conclusion, child linear growth, from the first 1000 d through school age, is positively associated with intellectual functioning and mental health of young school-aged children aged 6–7 y. Although early interventions in the first 2 y remain critical, interventions to improve nutritional status should consider various appropriate times during the life course, including preprimary and early primary school-age children. In addition to child-focused interventions, the high prevalence of infants born SGA or preterm suggests a need for earlier maternal-focused interventions as well to address growth faltering in utero. Our findings also suggest a possible association between higher BMI at age 2 y and greater mental health concerns in school-age children at age 6–7 y, which warrants further investigation. These findings are relevant and timely for many LMIC that are experiencing the nutrition transition and need to consider the implications of increases in the prevalence of overweight and obesity during the preschool years while continuing to address linear growth faltering during early childhood.

## Supplementary Material

nxab182_Supplemental_File

## Notes

Funding for this research was provided by the Nestlé Foundation, the Mathile Institute for the Advancement of Human Nutrition, and the Micronutrient Initiative. The funders had no role in study design, data collection and analysis, data interpretation, decision to publish, or preparation of the manuscript.

Author disclosures: The authors report no conflicts of interest.

PHN is a member of the Journal's Editorial Board.

Supplemental Figure 1 and Supplemental Tables 1 and 2 are available from the “Supplementary data” link in the online posting of the article and from the same link in the online table of contents at https://academic.oup.com/jn/.

Abbreviations used: BMIZ, body-mass-index *z*-score; FA, folic acid; FSIQ, full-scale intelligence quotient; HAZ, height-for-age *z*-score; HOME, Home Observation for Measurement of the Environment; IFA, iron and folic acid; LMIC, low- and middle-income countries; MM, multiple micronutrients; PRI, Perceptual Reasoning Index; PSI, Processing Speed Index; RCT, randomized placebo-controlled trial; SDQ, Strengths and Difficulties Questionnaire; SES, socioeconomic status; SGA, small for gestational age; VCI, Verbal Comprehension Index; WISC-IV, Wechsler Intelligence Scale for Children—Fourth Edition; WMI, Working Memory Index.
