# Supplementary material for: Child Linear Growth During and After the First 1000 Days Is Positively Associated with Intellectual Functioning and Mental Health in School-Age Children in Vietnam
Source: J Nutr. 2021 Jun 10;151(9):2816–24. doi: 10.1093/jn/nxab182 (PMC8417934; doi:10.1093/jn/nxab182)
Supplement: nxab182_Supplemental_File [file nxab182_supplemental_file.pdf]

Nguyen et al. (2021). Child linear growth during and after the first 1000 days is positively associated with intellectual functioning and mental health among school-age children in Vietnam
Online supporting materials

Supplemental Figure 1. Flow diagram of participant progress throughout the study (n=1,579 singleton live births)[1, 2]

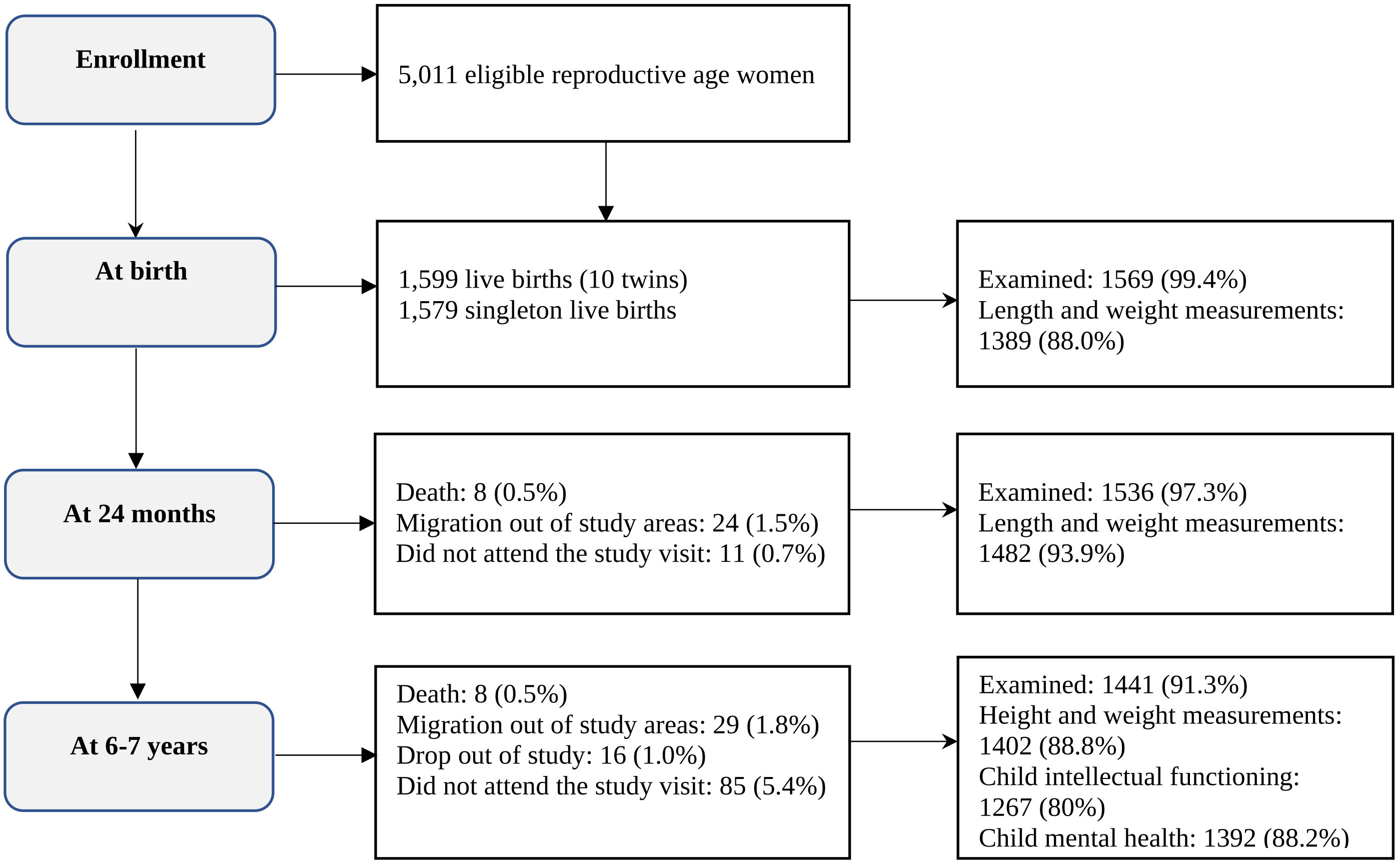

[1]Death and migration are cumulative. [2]All percentages were calculated using the total eligible birth sample (n=1579)

Nguyen et al. (2021). Child linear growth during and after the first 1000 days is positively associated with intellectual functioning and mental health among school-age children in Vietnam
Online supporting materials

Supplemental Table 1. Comparison of maternal and offspring characteristics between the final analytic sample and those missing outcome data at age 6-7 y[1, 2]

	**Analytic Sample** ***n = 1392***	**Excluded** ***n = 207***
	**Mean ± SD/ percent**	**Mean ± SD/ percent**
**Child characteristics**		
Child growth at 24 months		
Weight, *kg*	9.8 ± 1.0	9.6 ± 1.0
Length, *cm*	78.0 ± 2.7	77.5 ± 2.9
HAZ	-1.1 ± 0.9	-1.2 ± 1.0
BMIZ	-0.05 ± 0.8	-0.1 ± 0.8
Stunting, *%*[2]	16.4	23.3
Overweight/obese, *%*	9.6	8.8
Other child characteristics		
Female, *%*	49.5	48.3
Gestational age, *w*	39.2 ± 2.0	39.1 ± 2.1
Preterm, *%*	9.6	12.8
Low birth weight, *%*	4.8	4.7
SGA, *%*	15.3	18.2
Exclusive breastfeeding at 3 months, *%*	59.5	56.3
Dietary diversity score at 2y[2]	4.7 ± 1.2	4.3 ± 1.4
**Maternal characteristics**		
Age, *y*	25.9 ± 4.3	25.9 ± 4.4
Education, *%* [2]		
Primary school	7.5	13.8
Middle school	55.3	46.0
High school	25.6	23.0
College or higher	11.7	17.2
Preconception anemia, *%*	19.9	19.8
Depressive symptom (CESD>=10), *%*[2]	9.2	14.4
Maternal IQ, *score*	86.7 ± 16.9	85.9 ± 16.9
**Household characteristics**		
Socio-economic status, *%*		
Low	33.3	33.0
Average	33.9	29.5
High	32.8	37.6
Home environment at 1y, *score*	63.3 ± 8.2	63.4 ± 9.2

[1]Values are means ± SDs or percentages; [2]*P*<0.05 for comparisons between analytic sample and those excluded using Chi-square test for categorical variables and student T-test for continuous variables
BMIZ: Body-mass-index for-age z-score; CES-D: Center for Epidemiologic Studies Depression Scale; HAZ: Height-for-age Z-score; IQ: Intelligence Quotient; SGA: Small for gestational age.

Supplemental Table 2: Associations of child growth during the first 1000 days and beyond with child intellectual development and mental health at 6-7y (model considered growth velocity from birth to 2y)

Outcomes	FSIQ (n=1,267)		SDQ (n = 1,392)	
	Model 1	Model 2	Model 1	Model 2
	β (95% CI)	β (95% CI)	β (95% CI)	β (95% CI)
Birth length Z-score	-0.36 (-1.20, 0.48)	-0.17 (-1.14, 0.80)	-0.07 (-0.30, 0.16)	-0.11 (-0.37, 0.15)
Conditional height gain 0-2y	1.34 (0.57, 2.10)	0.96 (0.10, 1.81)	-0.09 (-0.30, 0.11)	-0.06 (-0.29, 0.16)
Conditional height gain 2-7y	1.12 (0.31, 1.93)	0.90 (-0.01, 1.82)	-0.27 (-0.48, -0.05)	-0.21 (-0.45, 0.03)
Birth weight Z-score	1.01 (0.03, 1.99)	0.57 (-0.54, 1.68)	0.17 (-0.09, 0.43)	0.19 (-0.09, 0.48)
Conditional weight gain 0-2y	-0.21 (-0.96, 0.54)	-0.40 (-1.24, 0.45)	0.18 (-0.02, 0.37)	0.13 (-0.09, 0.35)
Conditional weight gain 2-7y	0.00 (-0.72, 0.73)	-0.11 (-0.94, 0.71)	-0.07 (-0.27, 0.12)	-0.09 (-0.31, 0.13)

Model 1 adjusted for child age and sex; Model 2 adjusted for maternal (age, parity, and education) and child factors (age, sex, and pre-school education), home environment at 1y and 6-7y, household socioeconomic status and treatment group.
